# Isospectrally patterned lattices

**DOI:** 10.1038/s41598-025-13336-1

**Published:** 2025-07-29

**Authors:** Peter Schmelcher

**Affiliations:** 1 Zentrum für Optische Quantentechnologien, Fachbereich Physik, Universität Hamburg, Luruper Chaussee 149, 22761 Hamburg, Germany; 2 The Hamburg Centre for Ultrafast Imaging, Universität Hamburg, Luruper Chaussee 149, 22761 Hamburg, Germany; 3 ITAMP, Center for Astrophysics | Harvard & Smithsonian, 02138 Massachusetts, USA

**Keywords:** Quantum simulation, Theoretical physics

## Abstract

We introduce and explore patterned lattices consisting of coupled isospectral cells that vary across the lattice. The isospectrality of the cells is encapsulated in the phase that characterizes each cell and can be designed at will such that the lattice exhibits a certain phase gradient. Focusing on the specific example of a constant phase gradient on a given finite phase interval we show that the resulting band structure consists of three distinct energy domains with two crossover edges marking the transition from single center localized to delocalized states and vice versa. The characteristic localization length emerges due to a competition of the involved phase gradient on basis of a local rotation and the coupling between the cells which allows us to illuminate the underlying localization mechanism and its evolution. The fraction of localized versus delocalized eigenstates can be tuned by changing the phase gradient between the cells of the lattice. We outline the perspectives of investigation of this novel class of isospectrally patterned lattices.

## Introduction

Symmetries are ubiquituous in our description of quantum matter and represent a powerful means to analyze and classify its properties^1^. They define a unique starting-point for a subsequent deductive analytical or numerical study, a famous example being the theory of band structure which is based on Bloch’s theorem due to crystalline translation invariance^2,3^. The latter implies completely delocalized Bloch states. The absence of any symmetries in the case of disorder leads in one and two spatial dimensions to a spectrum of localized states^4,5^. Quasicrystals with their aperiodic long-range order fall into the substantial gap between these two limiting cases^6–15^. They show fractal energy spectra, critical localization of eigenstates, and arrange in so-called quasibands^16–20^. The coexistence of localized and delocalized eigenstates in aperiodic systems can involve a so-called mobility edge which marks the transition energy separating the different classes of states^21^ or can become manifest in an intermediate phase of interdispersed localized and delocalized states without mobility edge. The paradigm for exploring mixed localization-delocalization behavior in one spatial dimension is the Aubry-André quasiperiodic model^22^, whose modifications and generalizations have been extensively explored^23–39^ in particular in recent years. It should be noted that (infinitely extended) quasiperiodic or even disordered lattice models are homogeneous in the sense that their overall structure remains the same throughout the lattice, i.e. it does not depend on the region of the lattice.

Quasicrystals do not possess global symmetries but a plethora of local symmetries^15,40^. The impact of the presence of local symmetries in general settings, i.e. beyond the paradigm of quasicrystals, has been explored recently for both continuous and discrete one-dimensional systems^41–46^. Local symmetries allow to classify resonances in wave scattering^42,43^ and enhance the transfer efficiency in lattices^47^. Signatures of local symmetries have been observed experimentally in both lossy acoustic waveguides^48^ and coupled photonic wave guide lattices^49^. A typical spectral feature in the presence of local symmetries is the localization of eigenstates on the corresponding local symmetry specific domains of a given lattice (see in particular^40^). The underlying mechanism of this steered localization behavior has been identified^50^ as the isospectrality of the isolated symmetry-related subdomains i.e. applying a reflection or translation operation to a given lattice domain does not alter its eigenvalues. As a consequence we obtain pairwise degenerate eigenvalues that split linearly with an increasing coupling strength of these symmetry-related subdomains. This is the key ingredient to the present work: we elevate these properties to a working principle that generates a new category of lattices being composed of coupled isospectral cells, beyond the notion of local symmetries. The isospectral cells are parametrized by phase angles, or shortly, phases: for $K \times K$-cells there is $\frac{K(K-1)}{2}$ such phases. For isospectrally patterned lattices (IPL) these phases vary from cell to cell in a controlled manner across the lattice. While this opens the doorway of many possible such variations and resulting lattice setups we focus here, as a first exploration of an IPL, on cells characterized by a single phase. Our lattice covers a fixed phase interval by ’moving’ across the lattice. By definition, these specific IPL are finite and of inhomogeneous character. The aim of this work is to perform a first spectral analysis of these IPL on the basis of their eigenvalues and eigenstates. Each of the resulting energy bands is divided into three distinct branches. Two of those branches show single center (SC) localized states based on a characteristic localization length which results from the competition of the coupling and the (discrete) phase gradient relating different isospectral cells. They are separated by a finite system localization delocalization crossover (FLDC) from a third branch for which the delocalized eigenstates extend over the complete lattice. We determine the behavior of the fraction of (de-)localized states with varying phase gradient and coupling strength as well as number of lattice sites.

**Fig. 1:**
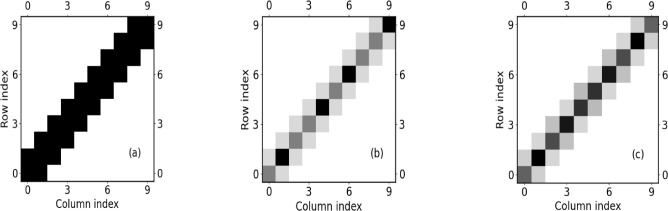
A gray scale sketch of the 10-site lattice tight-binding Hamiltonian for a (a) single site periodic (b) diagonal Fibonacci with constant off-diagonal coupling (c) an IPL chain. The change of the intracell off-diagonal along the chain while keeping the intercell off-diagonal coupling constant is clearly visible for the IPL chain. For both the periodic and the Fibonacchi setup the off-diagonal is constant throughout.

## Setup and Hamiltonian

According to^50^ it is the isospectrality of symmetry-related (isolated) subdomains and the resulting pairwise degeneracy of eigenvalues which underlie the observed localization of eigenstates on locally symmetric domains. Isospectrally patterned lattice go beyond the concept of local symmetries. They exploit the degree of freedom of isospectrality without any reference to the presence of a local symmetry. Therefore, generally speaking a key ingredient for the design of isospectrally patterned lattices are coupled cells all of which possess the same set of energy eigenvalues. The latter can be guaranteed by creating the cells as an orthogonal (or in general unitary) transformation ${\textbf{O}}_{\phi _m}$ of a given diagonal matrix ${\textbf{D}}$, i.e. we have for the $K \times K$ matrices ${\textbf{A}}_{m}$ of the cells that form the lattice the following appearance

1 $$\begin{aligned} {\textbf{A}}_{m} = {\textbf{O}}_{\phi _m}^{-1} {\textbf{D}} {\textbf{O}}_{\phi _m} \end{aligned}$$

where $m \in \{1,...,N\}$ stands for the cell index i.e. *N* is the number of cells of the lattice. ${\varvec{\phi }}_m$ represents the set of phase angles $\{\phi ^1_m,...,\phi ^{N_p}_m\}$, or shortly phases, that characterizes and parametrizes the $m-$th cell. The cells are represented by $K \times K$ matrices which contain $N_p=\frac{K(K-1)}{2}$ such phases, which are the phases of the most general orthogonal transformation in *K* dimensions.

The phases ${\varvec{\phi }}_m$ can in principle be chosen arbitrarily but designing the IPL according to some rule which generates a phase pattern along with varying sites of the lattice is a promising way to go. There is a large variety and flexibility in designing the phase sequences which adds to the richness of IPL. Finite lattices covering certain phase intervals for each of the phases in the set $\{\phi ^1_m,...,\phi ^{N_p}_m\}$ represent inhomogeneous setups whereas periodic or quasiperiodic setups are possible by correspondingly choosing the phase difference between neighboring cells being rational or irrational and, principally, extending the lattice size to infinity.

Our lattice Hamiltonian is composed of *N* cells and diagonal blocks ${\textbf{A}}_{m},m \in \{1,...,N\}$ coupled via off-diagonal blocks ${\textbf{C}}_{m}, m \in \{1,...,N-1\}$ and reads as follows

2 $$\begin{aligned} {\mathscr {H}}= & \sum _{m=1}^{N} \left( {|{m}\rangle } {\langle {m}|} \otimes \textbf{A}_{m} \right) \nonumber \\+ & \sum _{m=1}^{N-1} \left( {|{m+1}\rangle } {\langle {m}|} \otimes \textbf{C}_{m} + h.c. \right) \end{aligned}$$

The cell subspace could therefore be considered as internal degrees of freedom and the cell index as an external degree of freedom. $N_s$ will in the following denote the total number of lattice sites within and across cells. We will focus throughout this work on the case $K=2$ resulting in a single phase parameter $\phi$ which varies across the cells of the lattice. We will also focus throughout on finite lattices covering a finite interval of the phase $\phi$ and possessing a constant phase difference between neighboring cells. We employ open boundary conditions for our lattice.

The inhomogeneity of our lattice is implemented by our choice of the values $\phi _m$: we choose an equidistant grid of angles centered around the value $\frac{\pi }{4}$. This choice is motivated by the fact that the eigenvectors of ${\textbf{A}}_{\frac{\pi }{4}}$ are, independent of $\textbf{D}$, maximally delocalized in the $m-$th cell, providing a distinct starting-point for the control of localization versus delocalization on the lattice consisting of many coupled cells. The complete angular (or phase) range covered by the lattice reads then $[\frac{\pi }{4}-\frac{L}{2},\frac{\pi }{4}+\frac{L}{2}]$ with $L = \frac{\pi }{4} \cdot \frac{1}{L_f}$ where $L_f$ is a scaling factor of the phase range of the lattice, and we have $\phi _m = \frac{\pi }{4} - \frac{L}{2} + \frac{m-1}{N-1} L, m \in \{1,...,N\}$. Our lattice possesses, by construction, an inversion symmetry around its center $\phi = \frac{\pi }{4}$. We note, that for the limiting case $L_f \rightarrow \infty$ we obtain a (finite) periodic lattice with the unit-cell being ${\textbf{A}}_{\frac{\pi }{4}}$ whereas for $L_f = 1.0$ the lattice covers the angular range $[\frac{\pi }{8},\frac{3\pi }{8}]$.

Some further explanations and general remarks are in order. According to the above we have for a grid of a single phase ${\textbf{A}}_{m} = {\textbf{O}}_{{\phi }_m}^{-1} {\textbf{D}} {\textbf{O}}_{{\phi }_m}$ or written out explicitly this yields

3 $$\begin{aligned} \begin{aligned} \textbf{A}_m =&\begin{bmatrix} \cos \phi _m & \sin \phi _m \\ - \sin \phi _m & \cos \phi _m \\ \end{bmatrix} \begin{bmatrix} d_1 & 0 \\ 0 & d_2 \\ \end{bmatrix} \begin{bmatrix} \cos \phi _m & -\sin \phi _m \\ \sin \phi _m & \cos \phi _m \\ \end{bmatrix} \\ =&\begin{bmatrix} d_1 \cos ^2 \phi _m + d_2 \sin ^2 \phi _m & (d_2 - d_1) \sin \phi _m \cos \phi _m\\ (d_2 - d_1) \sin \phi _m \cos \phi _m & d_1 \sin ^2 \phi _m + d_2 \cos ^2 \phi _m \\ \end{bmatrix} \end{aligned} \end{aligned}$$

$\textbf{A}_m$ possesses the determinant $d_1 \cdot d_2$ and the trace $d_1 + d_2$. Employing the substitution $\phi _m = \psi _m + \frac{\pi }{4}$, which represents a shift of the phase, and inserting it into the above expression one obtains

4 $$\begin{aligned} \begin{aligned} \textbf{A}_m =&\frac{d_1+d_2}{2} {\mathbb {1}} + \frac{d_2-d_1}{2} \begin{bmatrix} \sin ( 2\psi _m) & \cos ( 2 \psi _m)\\ \cos ( 2\psi _m) & -\sin ( 2 \psi _m)\\ \end{bmatrix} \end{aligned} \end{aligned}$$

which splits $\textbf{A}_m$ into a part proportional to the identity matrix, i.e. a global offset, and a traceless contribution comprising its nontrivial part. The coupling between the cells (see eq.(2)) is provided by the $2 \times 2$ matrix ${\textbf{C}}={\textbf{C}}_m = \frac{\epsilon }{2} \left( \sigma _x + i \sigma _y \right)$ and we use open boundary conditions for our lattice. Here $\epsilon$ denotes the coupling strength and $\sigma _i, i=x,y$ are the $2 \times 2$ hermitian and unitary Pauli matrices. Our main focus will be on the weak to intermediate coupling regime $0< \epsilon < 0.5$. Importantly, neglecting the global offset, and extracting the prefactor $\frac{d_2-d_1}{2}$ (see eq.(4)) from the total lattice Hamiltonian it becomes evident that our Hamiltonian depends not on $\epsilon ,d_1,d_2$ separately but solely on the ratio $\frac{2\epsilon }{d_2-d_1}$. As a consequence we have focused in our numerical simulations on the variation of $\epsilon$. We emphasize that the phenomenology of the below-described numerical results persists with varying $\frac{2\epsilon }{d_2-d_1}$.

To provide a pictorial illustration of our IPL chain in comparison with a simple periodic tight-binding chain and a corresponding on-site Fibonacchi chain (ABAABABABAAB) we show in Fig.1 (a,b,c) via gray scale coding the Hamiltonian matrix for a lattice with ten sites for the mentioned three cases. While the periodic case shows complete uniformity, and the Fibonacchi chain possesses variations only on its diagonal entries, the IPL shows changes of the intracell off-diagonal coupling along the chain while keeping the intercell off-diagonal coupling constant.

We remark that the above-designed lattices represent one-dimensional chains but could straightforwardly be transfered to higher dimensions by introducing the corresponding off-diagonal couplings. It is important to note that the broad class of IPL contains several important more specialized models. The SSH-model (see ref^51^. and references therein) is obtained by making all phases across the lattice of equal value $\psi = \frac{\pi }{2}$ such that, after subtracting the global offset value, the diagonal entries vanish. The dimerized character stems then from the intracell coupling and the intercell coupling (see below). The (time-independent) Rice-Mele model is obtained for a constant but arbitrary phase $\psi$ (see^52^ and references therein).

For the following presentation of our results we should clarify our use of the notion of localization vs. delocalization. Localized states are (non-fragmented) states residing within our (finite) IPL and do not reach the boundaries of the lattice whereas delocalized states extend over the complete lattice. Equally we refer to the crossover point in energy where localized states turn into delocalized states as a FLDC edge.

**Fig. 2:**
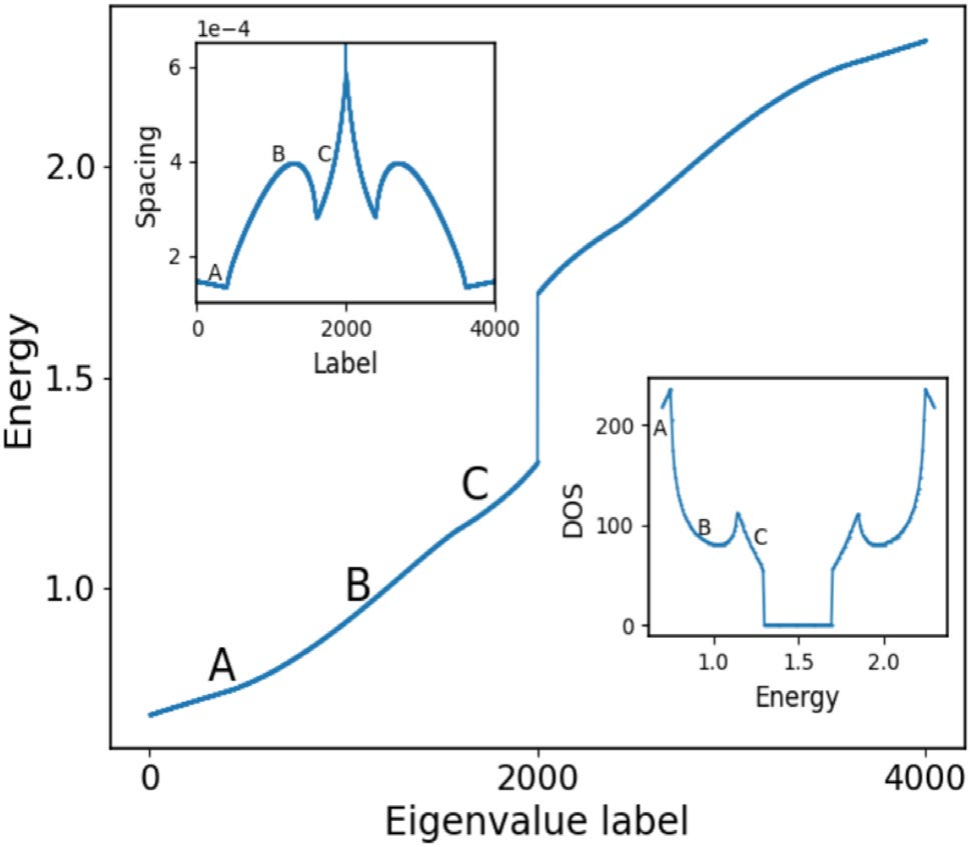
Main figure: energy eigenvalue spectrum of the equidistant $\phi$ lattice for $d_1=1,d_2=2, L_f=1.0, \epsilon =0.3, N_s=4002$, where $d_1,d_2$ are the diagonal values of ${\textbf{D}}$. Upper left inset: the corresponding energy level spacing. Lower right inset: the density of states for $N_s=16002$. We have chosen a larger value for $N_s$ to obtain a somewhat smoother behavior for the density of states with varying energy. The labels A,B,C mark the three distinct energetical regimes of the band which reflect itself correspondingly in the level spacing and the density of states.

## Phenomenology of the eigenvalue spectrum

Our approach is to numerically diagonalize the IPL Hamiltonian and analyze the resulting eigenvalues and eigenvectors. We first address the eigenvalue spectrum belonging to the Hamiltonian ${\mathscr {H}}$ in eq.(2) for an IPL with several thousand sites and $L_f=1.0$ for a coupling strength $\epsilon = 0.3$ and diagonal values $d_1=1,d_2=2$ of $\textbf{D}$, as shown in Fig.2. We observe two bands separated by a band gap (note, that we are using the terminology of a band, for reasons of convenience, although our setup is indeed non-periodic), our subsequent statements holding essentially for both bands. Each band can be divided into three distinct energy domains marked as A, B, C in Fig.2, which correspond to the lower, middle and upper energy domain of the lower band. Obviously, the energy eigenvalues with increasing degree of excitation show a prominent difference from the cosine-dispersion relation of the (monomer) periodic tight-binding case. In particular we witness close to the edges of the bands an approximately linear behavior of the energies. To work this out in more detail, the upper left inset in Fig.2 shows the spectrum of the eigenvalue spacing clearly exposing three domains with qualitatively different behavior for each band. While the region A shows a linearly decreasing spacing with a small variance, region B exhibits a highly nonlinear and nonmonotonic dependence with a large variance. Finally, region C displays a very peaked close to linear and therefore monotonous behavior with a large variance. The three domains can also be identified in the density of states shown as the lower right inset in Fig. 2. In region A we observe a high density of states which is even increasing within this domain and followed by a steep decline of the density of states in region B with partial recovery for higher energies, and finally, in region C, we observe an approximately linear decrease. This behavior persists qualitatively with varying coupling strength, noting that the energy gap between the two bands closes for $\epsilon = 0.5$. With increasing value of $L_f$ the covered phase interval shrinks and consequently the sizes of the domains A and B also shrink.

**Fig. 3:**
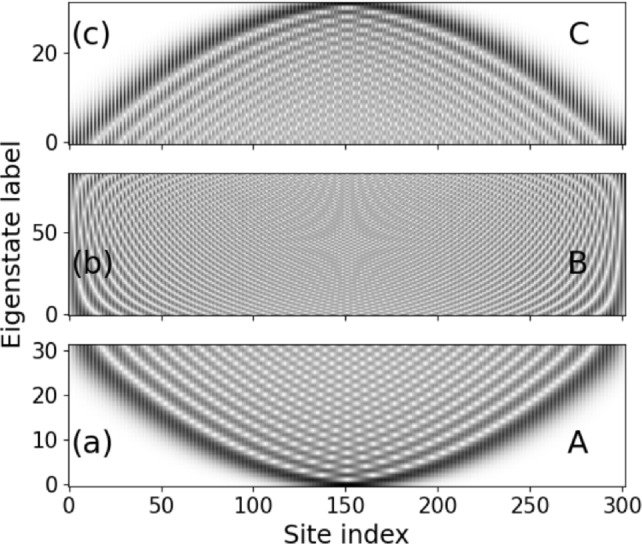
Grey scale eigenstate map showing the absolute values of all eigenstate components for the lower band of the spectrum of the lattice with $d_1=1,d_2=2, L_f=1.0, \epsilon =0.3, N_s=302$. Note that the grey scale has been renormalized for each eigenstate row. The sequence of eigenstates is divided into three domains (a,b,c) according to the energy domains A,B,C of the lower band in Fig.2. Note that the counting of the eigenstate labels is reset to zero within each domain providing a total of 151 eigenstate profiles.

## Eigenstate analysis: localized versus delocalized states

We have identified above three distinct domains within each energy band which we analyze now in terms of the behavior of their eigenstates. Fig.3 shows a greyscale eigenstate map, i.e. the magnitude of the eigenstate components, for the complete first band. The subfigures 3 (a,b,c) correspond to the domains A,B,C in the first energy band in Fig.2, respectively. We observe that in the domain A (Fig.3(a)) all eigenstates are localized around a single center and increasingly spread with increasing degree of excitation. In domain B the eigenstates are delocalized over the complete lattice, and, finally, in domain C localization takes over again and the eigenstates become increasingly SC localized with increasing degree of excitation. This behavior persists for varying coupling strength, in particular also for weak couplings, and for varying angular interval *L* (see below for quantitative statements) and is therefore of generic character. It repeats in the second upper band. We therefore encounter within each band two FLDC edges marking the transition from localized to delocalized eigenstates.

**Fig. 4:**
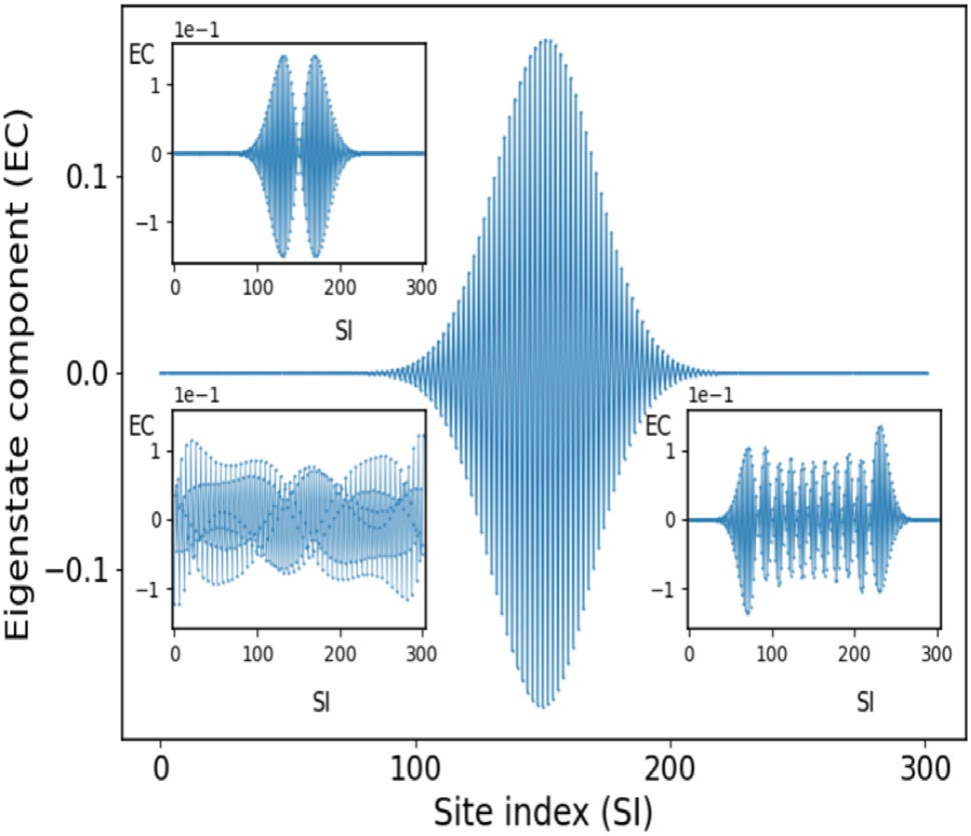
Localized states: the ground (main figure), first excited (top left inset) as well as tenth excited state (bottom right inset) of the first band in the domain A (see Figs.2,3). A delocalized state is shown for comparison (lower left inset) that belongs to domain B. Parameters are the same as in Fig.3.

Let us inspect the eigenstate profiles in some more detail with the aim to understand the origin of our observed SC localization. Fig. 4 shows, for the same parameter values as in Fig. 3, the ground state as well as the first and tenth excited states in the first band, thereby observing the increasing spreading of the eigenstates.

While our lattice consists of $N_s=302$ sites, the ground state shows a localization length of the order of 50 sites, whose origin and mechanism we shall analyze in the following. A closer inspection reveals that the envelope of the ground state is very well described by a Gaussian wave function. The fast oscillations from site to site can be attributed to the fact that ${\textbf{A}}_{\frac{\pi }{4}}$ possesses the eigenvectors $(1,-1),(1,1)$. Resultingly, a variational ansatz for the ground state wave function reads as follows

5 $$\begin{aligned} {|{\Psi }\rangle } = {\mathscr {N}} \sum _{n=1}^{N} \text {exp} \left( - \alpha \left( n-n_0 \right) ^2 \right) {|{n}\rangle } \otimes (1,-1) \end{aligned}$$

where ${\mathscr {N}} = \frac{1}{\sqrt{2}} \left( \sum _n {\text {exp}} \left( - 2 \alpha \left( n-n_0 \right) ^2 \right) \right) ^{-\frac{1}{2}}$ is the normalization constant and $\alpha$ is a variational parameter to be determined by minimizing the corresponding energy $E = {\langle {\Psi }|} {\mathscr {H}} {|{\Psi }\rangle }$. Evaluating this expectation value involves approximating the summations by continuous integrals and leads to the final result

6 $$\begin{aligned} E= & \frac{1}{2} \left( d_1 + d_2 \right) + \frac{1}{2} \left( d_1-d_2 \right) {\text {exp}} \left( - \frac{1}{4 \beta } \right) \nonumber \\ & - \hspace{0.1cm} \epsilon \hspace{0.1cm} {\text {exp}} \left( - \frac{\alpha }{2} \right) \end{aligned}$$

where $\beta = \frac{8 \alpha N^2}{\pi ^2}$. Note that $\frac{\pi }{4 N L_f}$ is the phase gradient across our lattice with $L_f = 1.0$ in the present case. The two competing second and third terms in the energy eq.(6) are due to the phase change across the cells on the diagonal and the off-diagonal coupling terms, respectively.

Varying $\alpha$ there exists a single minimum which amounts, for our specific case, to $\alpha _0 \approx 6.7 \cdot 10^{-3}$. The resulting energy agrees with the corresponding numerical value within one tenth of a per mill. The full width half maximum for these analytical considerations is 41 sites as compared to the numerical value of approximately 44 sites. The observed localization behavior therefore emerges in our lattice due to the competition in energy between the phase gradient among the isospectral cells and the coupling between the cells.

The eigenstates at the upper band edge can be obtained in a similar manner. From the above analysis, the position of the FLDC edge occurs at $n_{mob} \approx C \cdot \frac{N^2}{\sigma ^2}$ where $\sigma ^2$ is the variance of the Gaussian ground state and *C* being a constant of order one.

**Fig. 5:**
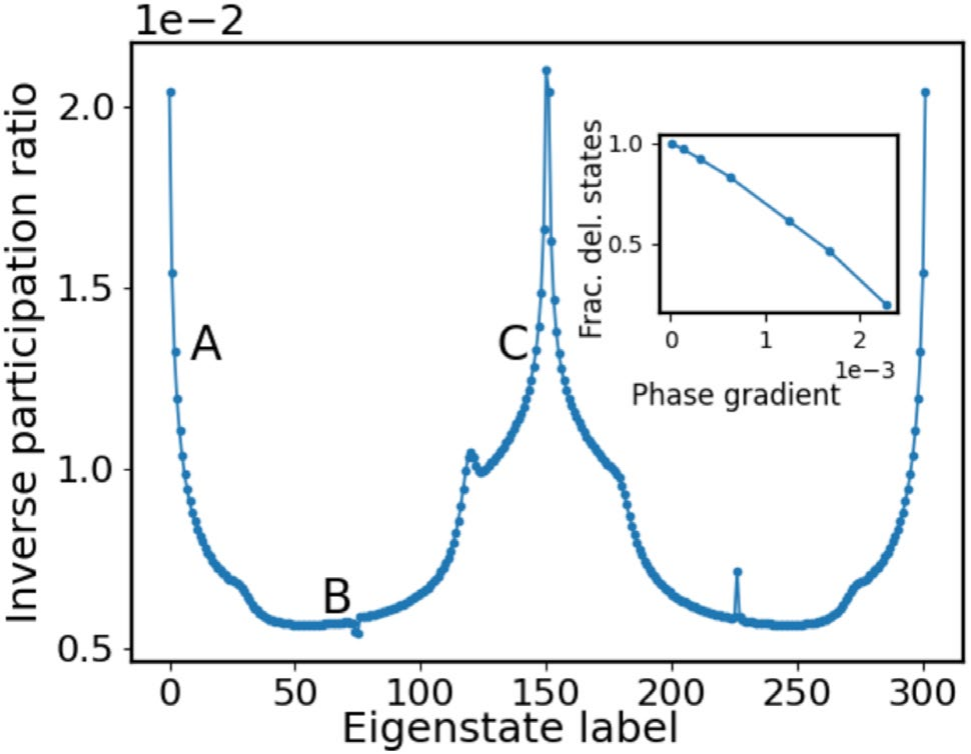
Main figure: The inverse participation ratio for all eigenstates across both bands. Parameters are the same as in Fig.3. Inset: The fraction of delocalized states with varying phase gradient for a fixed lattice size $N_s$. Labels A,B,C correspond to the different energy domains (see Fig. 2).

To quantify the degree of localization we determine the inverse participation ratio (IPR) for the complete spectrum of eigenstates, which is defined as $r = \sum _{i=1}^{N} |\psi _i|^4 \in [N^{-1},1]$. The maximal value for the IPR is one for an eigenvector localized on a single site of the chain and the minimal value $\frac{1}{N}$ is encountered for a state which is uniformly extended over the chain. As expected, see Fig.5, the IPR is large in the domains A and C of localized states whereas it is smallest in the regime B of delocalized states where a plateau of low values is encountered. According to the increasing delocalization in regime A with increasing degree of excitation the IPR peaks strongly for the ground state and low excitations but then rapidly decays when approaching the regime B. The reverse happens at the upper edge of the first band. A central moment analysis allows equally to distinguish between the different domains A,B and C with all odd moments being zero. It is instructive to compare the SC localization with the localization occuring for a random lattice for the same coupling strength $\epsilon = 0.3$ but random onsite binary disorder with the values corresponding to the eigenvalues of our IPL $2 \times 2$-sublattices, namely $d_1=1, d_2=2$. The IPR values of the random multi-centered eigenstates are typically significantly larger than 0.1 which has to be compared with the much smaller values of less than 0.02 for our IPL, see Fig. 5. This indicates the much larger localization length scale of the IPL SC localized eigenstates compared to the random lattice multi-centered eigenstates.

Some discussion concerning the fraction of SC localized vs. delocalized states is in order. For a sufficiently large value of $L_f$ practically all eigenstates are delocalized. With a decreasing value of $L_f$ for a fixed lattice size, i.e. an increasing phase gradient, we observe an approximately linear decrease of the fraction of delocalized states, see the inset of Fig.5. For e.g. $L_f=1.0$ corresponding to a phase gradient of $1.2 \cdot 10^{-3}$ (for the above other parameter values) 40 $\%$ of the eigenstates become SC localized. The fraction of (de-)localized states is independent of the coupling strength $\epsilon$. While our simulations naturally address finite lattices there is a systematic way of increasing the number of lattice sites $N_s$. Within this procedure we keep the coupling constant $\epsilon$ and in particular the covered total angular interval *L* fixed and step by step increase the number of lattice points $N_s$. It should be noted that this course of action maintains the inhomogeneous character of our lattice and might be reminescent of a continuum limit which should be carefully distinguished from a systematic increase of the lattice size in the case of homogeneous systems. We hereby observe that the fraction of (de-)localized states is independent of the lattice size which we have verified by varying $N_s$ over three orders of magnitude. For delocalized eigenstates close to the band center in regime *B* we obtain for the IPR $r \propto N_s^{-1}$, as expected, where as for localized states close to the band edges in regimes *A*, *C* we observe $r \propto N_s^{-0.5}$. Averaging over the eigenstates within the (localized) regimes *A*, *C* yields a scaling behavior according to $r_m \propto N_s^{-0.84}$ whereas in the (delocalized) regime *B* one obtains the well-known $r_m \propto N_s^{-1}$ scaling, where $r_m$ is the corresponding mean IPR. Our crossover from localized to delocalized states is robust against disorder, both for the coupling and for the eigenvalues in the isospectral cells, up to the several percent level, from which on localized structural changes in the eigenstates are manifest.

## Conclusions and perspectives

IPLs open a new pathway of systematically exploring and controlling a designed mixture and crossover between SC localized and delocalized states as well as devising the corresponding finite system localization delocalization crossover edges. The underlying mechanism of the (de-)localization is the competition between the underlying phase gradient across different isospectral cells of the lattice and the coupling among those cells. While we have been focusing here on an inhomogeneous IPL setup which consists of a grid of equidistantly separated phases covering a phase interval of the order of a single period, i.e. of the order of $2 \pi$, there is a plethora of different possibilities to design other isospectrally patterned lattices. For example, fixing the phase gradient and increasing the number of lattice sites such that the phase interval covers several periods or even extending it to infinity, establishes interesting IPL with potential novel properties depending, e.g., on the rationality of the phase gradient. Another future line of investigation would be to study the topological properties of IPL with varying coupling strength between the cells. A non-constant phase gradient across the lattice would add to this richness of setups. Going beyond rotations in two dimensions i.e. shaping the isospectral cells in the higher dimensional angular space intriguing phase structures will represent an interesting and promising avenue to be pursued.

Experimental platforms that might be suited to realize IPLs could be integrated photonic waveguide lattices^53,54^ or optical lattice/tweezer-based ultracold atomic systems which offer an astounding control of both external as well internal atomic degrees of freedom^55,56^. Specifically the recently established synthetic lattices of laser-coupled atomic momentum modes^57^ could be promising candidates for the realization of IPL where injection spectroscopy^58^ is available for probing energy spectra.

## Data Availability

Data sets generated during the current study are available from the corresponding author on reasonable request. By writing to the authors the spectral properties, eigenvalues and eigenvectors of the considered lattices can be obtained.
